# Supplementary material for: A Likelihood Approach for Real-Time Calibration of Stochastic Compartmental Epidemic Models
Source: PLoS Comput Biol. 2017 Jan 17;13(1):e1005257. doi: 10.1371/journal.pcbi.1005257 (PMC5240920; doi:10.1371/journal.pcbi.1005257)
Supplement: S1 File — (TAR.GZ) [file pcbi.1005257.s014.tar.gz › HSPH_Online-SI-Revision/EnKF07/n1-severe/n1-severe_PF2_EnKF-summary2.pdf]

| peak −8 | 3w spec | 3w cum | 5w spec | 5w cum | end | 3w spec LOG | 3w cum LOG | 5w spec LOG | 5w cum LOG | end LOG |
|---|---|---|---|---|---|---|---|---|---|---|
| mean | 341.8 | 886.6 | 96.1 | 324.7 | 61.9 | 0.8 | 0.8 | 1.2 | 0.6 | 0.5 |
| median | 169. | 611.8 | 90.1 | 277.7 | 57.9 | 0.7 | 0.7 | 1.1 | 0.6 | 0.4 |
| quantile .1 | 86.4 | 122.9 | 72.4 | 99. | 41.8 | 0.5 | 0.4 | 0.7 | 0.3 | 0.2 |
| quantile .9 | 707.2 | 1683.6 | 127.3 | 645.6 | 84.9 | 1.1 | 1.2 | 2. | 0.3 | 0.9 |
| 42 | 21 | 29 | 27 | 38 | | | | | | |

| peak −4 | 3w spec | 3w cum | 5w spec | 5w cum | end | 3w spec LOG | 3w cum LOG | 5w spec LOG | 5w cum LOG | end LOG |
|---|---|---|---|---|---|---|---|---|---|---|
| mean | 90.1 | 124.9 | 91.4 | 91.6 | 78.8 | 1.2 | 0.5 | 1.8 | 0.6 | 0.8 |
| median | 91.2 | 108.3 | 93.3 | 92.4 | 84.9 | 1.3 | 0.5 | 1.9 | 0.7 | 1. |
| quantile .1 | 73.8 | 85.5 | 81.3 | 64.1 | 60. | 0.6 | 0.3 | 1.3 | 0.3 | 0.4 |
| quantile .9 | 104.6 | 171.7 | 97.7 | 108. | 91.2 | 1.6 | 0.8 | 2.1 | 0.3 | 1.2 |
| 22 | 38 | 2 | 35 | 23 | | | | | | |

| peak | 3w spec | 3w cum | 5w spec | 5w cum | end | 3w spec LOG | 3w cum LOG | 5w spec LOG | 5w cum LOG | end LOG |
|---|---|---|---|---|---|---|---|---|---|---|
| mean | 98.6 | 109.8 | 96.9 | 100.5 | 92.4 | 1.4 | 0.7 | 1.8 | 0.8 | 0.9 |
| median | 94.2 | 107.6 | 95.2 | 99.3 | 91. | 1.3 | 0.7 | 1.8 | 0.8 | 1. |
| quantile .1 | 87.7 | 86.3 | 91.8 | 87.5 | 86.9 | 1. | 0.5 | 1.5 | 0.6 | 0.7 |
| quantile .9 | 110. | 129.3 | 102.2 | 113.1 | 98.7 | 1.6 | 0.8 | 2. | 0.6 | 1.1 |
| 15 | 31 | 3 | 31 | 21 | | | | | | |

| peak +4 | 3w spec | 3w cum | 5w spec | 5w cum | end | 3w spec LOG | 3w cum LOG | 5w spec LOG | 5w cum LOG | end LOG |
|---|---|---|---|---|---|---|---|---|---|---|
| mean | 547.8 | 161.4 | 475.9 | 154.3 | 144. | 1.1 | 0.5 | 1.4 | 0.6 | 0.7 |
| median | 114.3 | 131.5 | 102.1 | 124.5 | 106.2 | 1. | 0.5 | 1.4 | 0.6 | 0.7 |
| quantile .1 | 87.6 | 95.6 | 93. | 91.1 | 87.9 | 0.7 | 0.4 | 0.9 | 0.4 | 0.5 |
| quantile .9 | 170. | 202.2 | 270.5 | 192.1 | 166. | 1.5 | 0.7 | 1.9 | 0.4 | 1. |
| 34 | 47 | 24 | 45 | 40 | | | | | | |

| peak +8 | 3w spec | 3w cum | 5w spec | 5w cum | end | 3w spec LOG | 3w cum LOG | 5w spec LOG | 5w cum LOG | end LOG |
|---|---|---|---|---|---|---|---|---|---|---|
| mean | 1688.8 | 7450.2 | 865.4 | 8169.1 | 8741.7 | 1. | 0.7 | 1.2 | 0.7 | 0.8 |
| median | 160.2 | 266.3 | 146.9 | 212.2 | 169.4 | 1. | 0.5 | 1.2 | 0.5 | 0.6 |
| quantile .1 | 95.8 | 110.9 | 98. | 109.6 | 96. | 0.7 | 0.4 | 0.6 | 0.4 | 0.4 |
| quantile .9 | 10 210. | 1340.1 | 2748. | 1339.8 | 1677. | 1.4 | 0.8 | 1.8 | 0.4 | 1.1 |
| 43 | 41 | 31 | 38 | 37 | {} | {} | {} | {} | {} | {} |
